# Synthesis, bioactivities and phloem uptake of dipeptide-chlorantraniliprole derivatives

**DOI:** 10.1186/s13065-020-00673-7

**Published:** 2020-03-30

**Authors:** Shijie Zheng, Xiaomin Lin, Hanxiang Wu, Chen Zhao, Hanhong Xu

**Affiliations:** 1 State Key Laboratory for Conservation and Utilization of Subtropical Argo-Bioresources, South China Agricultural University, Guangzhou, Guangdong China; 2 Key Laboratory of Natural Pesticide and Chemical Biology, Ministry of Education, South China Agricultural University, Guangzhou, 510642 China

**Keywords:** Chlorantraniliprole, Phloem-mobile pesticides, Dipeptide, Insecticidal activity

## Abstract

Phloem systemicity is a desirable property for insecticides to control sucking insects. However, the development of phloem systemic insecticides is challenging. One possible strategy is to link existed insecticides with endogenous substances so that the resulting conjugates can be transported by specific transporters into the phloem. In this study, novel dipeptide promoieties were introduced into chlorantraniliprole, which is an efficient and broad-spectrum anthranilic diamide insecticide without phloem mobility. Twenty-two new dipeptide-chlorantraniliprole conjugates have been synthesized. Systemic tests showed that all conjugates exhibited phloem mobility in *Ricinus communis*. In particular, compound **4g** with alanyl-alanine dipeptide fragment was able to accumulate in phloem sap (114.49 ± 11.10 μM) in the form of its hydrolysis product **5g**. Results of bioassay showed that conjugates **4g** and **5g** were able to exhibit comparable insecticidal activity against *Plutella xylostella* L. and *Spodoptera exigua* compared to its parent compound chlorantraniliprole. This work demonstrated that the dipeptide structures were able to contribute to the improvement of the uptake and phloem mobility of chlorantraniliprole, and two phloem mobile conjugates with satisfactory in vivo insecticidal effect was obtained as new candidates for high-efficient insecticides.

## Introduction

With increasing demand for food and environmental safety, the use of pesticides is subjected to stringent restrictions. Furthermore, it was apparent to all that only a very small part of applied pesticide actually reached the sites of action, and the off-target portion became environmental pollutant [1, 2] which led to great public concern. Hence, the accurate and efficient utilization of agrochemicals was the focus of our research.

It has been demonstrated that coupling existing non-phloem mobile pesticide structures with endogenous substances, such as amino acids and saccharides, was effective on improving their phloem mobility [3–14]. For example, a series of phloem mobile glucose–fipronil conjugates (GTF and GOF), glycinergic–fipronil conjugate (GlyF) and alanine ester-chlorantraniliprole conjugate were synthesized in our previous work, and their uptake process was proven to be mediated by active transport systems [5, 6, 8]. Such carrier-mediated transport strategy has been considered as a promising way for vectorizing agrochemicals, which can enhance bioavailability of pesticides [15].

Dipeptides and tripeptides can be transported into plant cells through peptide transporters, which were first discovered in *Arabidopsis* plants [16] as a H^+^-coupled transporter for oligopeptides [17, 18]. Similar facilitator transporters, such as oligopeptide transporter (PepT1 and PepT2), were also found in mammals to drive the uptake of di- and tri-peptides [19, 20]. It has been proved that dipeptides could be used as promoieties to link with pharmacologically active substances and deliver prodrugs to target tissues [21–23]. Therefore, conjugating dipeptide structures with existing pesticides is expected to be another potential strategy to obtain new candidates for phloem-mobile pesticides, which could enhance the efficiency and reduce the consumption of pesticides.

## Materials and methods

### Chemistry

**Scheme 1:**
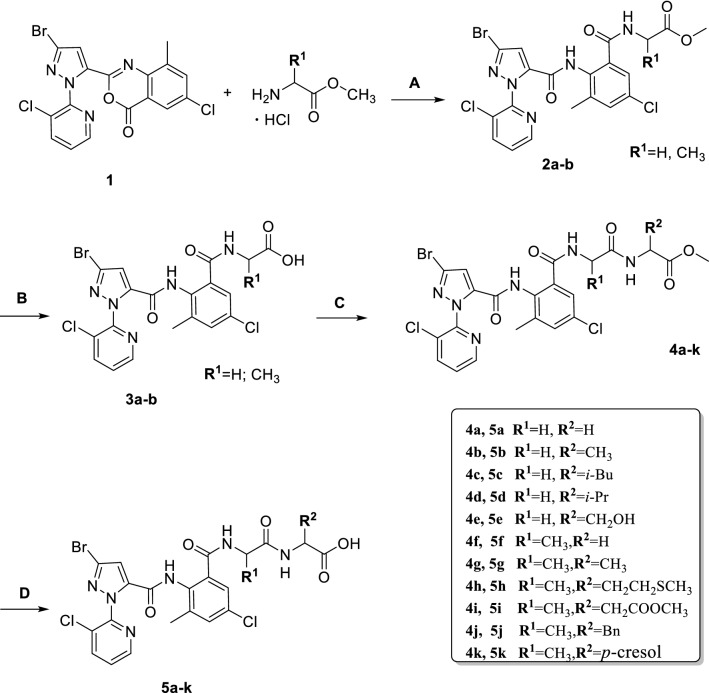
Synthetic route of target compounds **4a–k** and **5a–k**. General reaction conditions: **a** TEA, CH_2_Cl_2_, room temperature to 45 °C, 8 h; **b** LiOH, THF/H_2_O, 0 °C to room temperature, 2 h; **c** NMM, EDCI, DMAP, CH_2_Cl_2_, room temperature, overnight; **d** LiOH, THF/H_2_O, 0 °C to room temperature, 2 h

A series of new dipeptide-chlorantraniliprole conjugates were synthesized by coupling chlorantraniliprole with different dipeptides as shown in Scheme 1. Reacting intermediate **1** with corresponding amino acid methyl ester hydrochlorides led to **2a**–**b** [24, 25]. Then, to a solution of compound **2a** or **2b** in tetrahydrofuran (THF), lithium hydroxide was slowly added. After being stirred for 2 h, the reaction mixture was acidified to pH = 3 with 1 M hydrochloric acid (HCl), and the organic phase was concentrated under reduced pressure to obtain **3a** and **3b** [26]. To a solution of **3a** or **3b** in dry dichloromethane were sequentially added amino acid hydrochlorides, 1-(3-dimethylaminopropyl)-3-ethylcarbodiimide hydrochloride (EDCI), and *N*, *N*-dimethyl-4-aminopyridine (DMAP). The mixture was stirred at room temperature overnight. After evaporating the solvent in vacuo, the residue was purified by flash column chromatography (silica gel, petroleum ether/ethyl acetate = 2:1, v/v) to afford compounds **4a**–**k** [27]. Compounds **5a**–**k** were prepared with similar hydrolysis reactions as used to prepare compounds **3a** and **3b**. Purifications of the crude products were performed via flash column chromatography (silica gel, petroleum ether/ethyl acetate/acetic acid = 1:1:0.005, v/v/v).

Reagents were purchased from commercial resources and were used without further purification unless otherwise stated. All reactions were carried out under nitrogen atmosphere with dry solvents and were monitored by thin-layer chromatography (TLC) analysis using silica gel GF254 thin-layer plates, and spots were visualized with a ZF-20D ultraviolet (UV) analyzer. Column chromatography purification was performed over silica gel (200–300 mesh, Qingdao Marine Chemical Ltd.). ^1^H fourier transform nuclear magnetic resonance (FT-NMR) spectra were measured with a Bruker AV-600 MHz instrument and calibrated using residual un-deuterated solvents as internal references (CDCl_3_: *δ *= 7.26 ppm for ^1^H NMR; DMSO-*d*_*6*_: *δ *= 2.50 ppm for ^1^H NMR) [28]. Multiplicities were reported as follows: singlet (s), doublet (d), triplet (t), quartet (q), doublet of doublets (dd), and multiplet (m). Coupling constants were reported as *J* values in hertz. MS data were obtained using the Waters SYNAPT™ mass spectrometry.

#### General procedure for synthesis of compounds 2a–b

To a dry round-bottomed flask containing a solution of dry triethylamine (12 mmol) in 20 mL anhydrous dichloromethane (CH_2_Cl_2_), glycine methyl ester hydrochloride (1.50 g, 12 mmol) or l-alanine methyl ester hydrochloride (1.67 g, 12 mmol) was added under nitrogen, respectively. The reaction mixture was stirred for 30 min at room temperature. A solution of compound **1** (10 mmol) in anhydrous CH_2_Cl_2_ was then added dropwise under nitrogen. The suspension was warmed up to 45 ℃ and stirred for 8 h until TLC indicated complete consumption of the starting materials. The reaction mixture was cooled to room temperature, followed by the addition of water (10 mL). The mixture was extracted with ethyl acetate (3 × 10 mL). The combined organic layer was dried over Na_2_SO_4_, filtered and concentrated by rotary evaporation to obtain **2a** and **2b**, which were used in the next step without purification.

#### General procedure for synthesis of compounds 3a–b [29]

Compound **2a** or **2b** (5 mmol) was added in the mixed solvent of THF and H_2_O at 0℃, then lithium hydroxide (0.63 g, 15 mmol) was slowly added. After being stirred for 2 h, the reaction mixture was acidified to pH = 2 with HCl (1 M). The organic phase was removed by rotary evaporation. The residual aqueous solution was extracted with ethyl acetate (3 × 20 mL), and the combined organic extract was washed with H_2_O (3 × 20 mL), dried over Na_2_SO_4_, then concentrated under reduced pressure to obtain crude product of **3a** and **3b** which was used in the next step directly.

#### General procedure for synthesis of compounds 4a–k [27]

To a solution of **3a** or **3b** (10 mmol) in dry CH_2_Cl_2_ (10 mL) were sequentially added amino acid hydrochlorides (13 mmol), EDCI (2.88 g, 15 mmol), and DMAP (0.61 g, 5.0 mmol). The mixture was stirred at room temperature for overnight, then diluted with H_2_O (10 mL), acidified with citric acid (10%) and extracted with CH_2_Cl_2_ (3 × 10 mL). The combined organic layers were washed with saturated NaHCO_3_ aq. Solution and dried over Na_2_SO_4_, filtered, and concentrated. The residue was purified by flash column chromatography (silica gel, petroleum ether/ethyl acetate = 2:1, v/v) to afford compounds of **4a**–**k**.

##### *Methyl{2*-*[3*-*bromo*-*1*-*(3*-*chloropyridin*-*2*-*yl)*-*1H*-*pyrazole*-*5*-*carboxamido]*-*5*-*chloro*-*3*-*methylbenzoyl)glycylglycinate* (4a)

White solid in 79% yield; ^1^H NMR (600 MHz, DMSO-*d*_6_): *δ* 10.26 (s, 1H), 8.66 (s, 1H), 8.47 (dd, *J* = 4.7, 1.5 Hz, 1H), 8.19 (t, *J* = 6.0 Hz, 1H), 8.15 (dd, *J* = 8.1, 1.5 Hz, 1H), 7.59 (dd, *J* = 8.1, 4.7 Hz, 1H), 7.51 (d, *J* = 2.4 Hz, 1H), 7.49 (s, 1H), 7.31 (s, 1H), 3.83 (d, *J* = 5.9 Hz, 2H), 3.77 (d, *J* = 5.8 Hz, 2H), 3.62 (s, 3H), 2.16 (s, 3H). ^13^C NMR (151 MHz, DMSO-*d*_6_): *δ* 172.55, 172.48, 172.31, 165.79, 165.72, 156.03, 148.84, 147.53, 139.68, 139.16, 136.15, 131.77, 131.41, 128.33, 127.22, 127.04, 126.31, 111.14, 61.84, 54.96, 49.05, 18.11. ESI-HRMS calcd. For C_22_H_18_BrCl_2_N_6_O_5_: [M−H]^−^ 594.9899, found 594.9893.

##### *Methyl{2*-*[3*-*bromo*-*1*-*(3*-*chloropyridin*-*2*-*yl)*-*1H*-*pyrazole*-*5*-*carboxamido)*-*5*-*chloro*-*3*-*methylbenzoyl)glycyl*-*l*-*alaninate* (4b)

White solid in 71% yield; ^1^H NMR (600 MHz, DMSO-*d*_*6*_): *δ* 10.24 (s, 1H), 8.54 (t, *J* = 5.8 Hz, 1H), 8.49 (d, *J* = 4.6 Hz, 1H), 8.31 (d, *J* = 6.9 Hz, 1H), 8.16 (d, *J* = 7.9 Hz, 1H), 7.60 (dd, *J* = 8.2, 4.7 Hz, 1H), 7.49 (d, *J* = 15.7 Hz, 2H), 7.34 (s, 1H), 4.31 (q, *J* = 7.2 Hz, 1H), 3.85–3.73 (m, 2H), 3.62 (s, 3H), 2.16 (s, 3H), 1.27 (d, *J* = 7.2 Hz, 3H). ^13^C NMR (151 MHz, DMSO-*d*_*6*_): *δ* 173.11, 167.81, 167.62, 155.99, 148.79, 146.78, 139.13, 139.08, 138.41, 133.56, 132.17, 132.11, 130.59, 128.87, 128.09, 125.69, 125.05, 110.88, 52.68, 48.37, 43.26, 18.96, 18.14. ESI-HRMS calcd. For C_23_H_20_BrCl_2_N_6_O_5_: [M−H]^−^ 609.0056, found 609.0053.

##### *Methyl{2*-*[3*-*bromo*-*1*-*(3*-*chloropyridin*-*2*-*yl)*-*1H*-*pyrazole*-*5*-*carboxamido)*-*5*-*chloro*-*3*-*methylbenzoyl)glycyl*-*l*-*leucinate* (4c)

White solid in 65% yield; ^1^H NMR (600 MHz, DMSO-*d*_6_): *δ* 10.26 (s, 1H), 8.53 (t, *J* = 5.9 Hz, 1H), 8.49 (d, *J* = 4.7 Hz, 1H), 8.28 (d, *J* = 7.8 Hz, 1H), 8.16 (d, *J* = 8.0 Hz, 1H), 7.60 (dd, *J* = 8.1, 4.7 Hz, 1H), 7.50–7.46 (m, 2H), 7.34 (s, 1H), 4.32 (ddd, *J* = 9.8, 7.8, 5.1 Hz, 1H), 3.83 (dd, *J* = 16.6, 6.0 Hz, 1H), 3.76 (dd, *J* = 16.6, 5.8 Hz, 1H), 3.61 (s, 3H), 2.16 (s, 3H), 1.63–1.51 (m, 2H), 1.52–1.47 (m, 1H), 0.86 (d, *J* = 6.5 Hz, 3H), 0.83 (d, *J* = 6.4 Hz, 3H). ^13^C NMR (151 MHz, DMSO-*d*_6_): *δ* 173.30, 169.24, 166.51, 156.21, 148.70, 147.54, 139.88, 139.77, 139.29, 135.67, 131.99, 131.40, 128.15, 127.29, 127.02, 126.16, 111.20, 52.34, 50.73, 42.55, 40.42, 24.65, 23.16, 21.79, 18.15. ESI-HRMS calcd. For C_26_H_26_BrCl_2_N_6_O_5_: [M−H]^−^ 651.0525, found 651.0514.

##### *Methyl{2*-*[3*-*bromo*-*1*-*(3*-*chloropyridin*-*2*-*yl)*-*1H*-*pyrazole*-*5*-*carboxamido)*-*5*-*chloro*-*3*-*methylbenzoyl)glycyl*-*l*-*valinate* (4d)

White solid in 58% yield; ^1^H NMR (600 MHz, DMSO-*d*_*6*_): *δ* 9.99 (s, 1H), 8.41 (dd, *J* = 4.7, 1.6 Hz, 1H), 7.82 (dd, *J* = 8.1, 1.6 Hz, 1H), 7.36–7.32 (m, 2H), 7.27 (d, *J* = 1.7 Hz, 1H), 7.23 (t, *J* = 5.2 Hz, 1H), 7.03 (s, 1H), 6.67 (d, *J* = 8.7 Hz, 1H), 4.53 (dd, *J* = 8.7, 5.0 Hz, 1H), 4.04 (dd, *J* = 7.2, 5.2 Hz, 2H), 3.74 (s, 3H), 2.18 (s, 3H), 2.14 (dd, *J* = 11.9, 6.9 Hz, 1H), 0.91 (d, *J* = 6.9 Hz, 6H). ^13^C NMR (151 MHz, DMSO-*d*_6_): *δ* 172.90, 172.39, 165.68, 156.03, 148.83, 147.53, 139.69, 139.17, 135.98, 131.84, 131.38, 128.31, 127.22, 127.04, 126.33, 111.11, 57.55, 52.18, 49.18, 30.61, 19.47, 18.75, 18.52, 18.12. ESI-HRMS calcd. For C_25_H_24_BrCl_2_N_6_O_5_: [M−H]^−^ 637.0369, found 637.0359.

##### *Methyl{2*-*[3*-*bromo*-*1*-*(3*-*chloropyridin*-*2*-*yl)*-*1H*-*pyrazole*-*5*-*carboxamido)*-*5*-*chloro*-*3*-*methylbenzoyl)glycyl*-*l*-*serinate* (4e)

White solid in 64% yield; ^1^H NMR (600 MHz, DMSO-*d*_6_): *δ* 10.24 (s, 1H), 8.52 (t, *J* = 6.1 Hz, 1H), 8.49 (d, *J* = 4.7 Hz, 1H), 8.28 (d, *J* = 7.8 Hz, 1H), 8.16 (d, *J* = 8.0 Hz, 1H), 7.61 (dd, *J* = 7.3, 4.2 Hz, 1H), 7.50 (s, 1H), 7.44 (s, 1H), 7.36 (s, 1H), 5.09 (t, *J* = 5.7 Hz, 1H), 4.39 (q, *J* = 5.3 Hz, 1H), 3.85 (qd, *J* = 16.7, 5.9 Hz, 2H), 3.71 (dt, *J* = 11.0, 5.4 Hz, 1H), 3.64 (s, 3H), 3.63–3.59 (m, 1H), 2.16 (s, 3H). ^13^C NMR (151 MHz, DMSO-*d*_*6*_): *δ* 170.89, 168.90, 168.11, 156.37, 148.71, 146.63, 139.37, 139.00, 138.35, 133.33, 132.29, 131.69, 131.29, 129.07, 125.88, 125.38, 111.16, 62.13, 54.69, 52.81, 43.45, 29.68, 18.76. ESI-HRMS calcd. For C_23_H_20_BrCl_2_N_6_O_5_: [M−H]^−^ 625.0005, found 625.0007.

##### *Methyl{2*-*[3*-*bromo*-*1*-*(3*-*chloropyridin*-*2*-*yl)*-*1H*-*pyrazole*-*5*-*carboxamido)*-*5*-*chloro*-*3*-*methylbenzoyl)*-*l*-*alanylglycinate* (4f)

White solid in 76% yield; ^1^H NMR (600 MHz, DMSO-*d*_6_): *δ* 10.24 (s, 1H), 8.49 (s, 2H), 8.19 (s, 1H), 8.16 (dd, *J* = 8.1, 1.5 Hz, 1H), 7.61 (dd, *J* = 8.1, 4.7 Hz, 1H), 7.52 (dd, *J* = 2.5, 0.7 Hz, 2H), 7.35 (s, 1H), 4.31 (s, 1H), 3.82 (d, *J* = 5.9 Hz, 2H), 3.63 (s, 3H), 2.18 (s, 3H), 1.21 (d, *J* = 7.2 Hz, 3H). ^13^C NMR (151 MHz, DMSO-*d*_6_): *δ* 173.06, 170.59, 166.07, 156.12, 148.80, 147.54, 139.77, 139.71, 138.92, 135.97, 131.80, 131.75, 131.33, 128.27, 127.25, 127.05, 126.42, 111.10, 52.14, 49.23, 41.00, 18.11, 18.07. ESI-HRMS calcd. For C_23_H_20_BrCl_2_N_6_O_5_: [M−H]^−^ 609.0056, found 609.0042.

##### *Methyl{2*-*[3*-*bromo*-*1*-*(3*-*chloropyridin*-*2*-*yl)*-*1H*-*pyrazole*-*5*-*carboxamido)*-*5*-*chloro*-*3*-*methylbenzoyl)*-*l*-*alanyl*-*l*-*alaninate* (4g)

White solid in 76% yield; ^1^H NMR (600 MHz, DMSO-*d*_6_): *δ* 10.25 (s, 1H), 8.49 (dd, *J* = 4.7, 1.6 Hz, 1H), 8.37 (d, *J* = 4.9 Hz, 1H), 8.27 (d, *J* = 7.4 Hz, 1H), 8.16 (dd, *J* = 8.1, 1.5 Hz, 1H), 7.60 (dd, *J* = 8.1, 4.7 Hz, 1H), 7.50 (dd, *J* = 7.3, 2.3 Hz, 2H), 7.36 (s, 1H), 7.36 (s, 1H), 4.39–4.32 (m, 1H), 4.31–4.25 (m, *J* = 7.3, 2.7 Hz, 1H), 3.62 (s, 3H), 2.18 (s, 3H), 1.26 (d, *J* = 7.3 Hz, 3H), 1.21 (d, *J* = 7.2 Hz, 3H). ^13^C NMR (151 MHz, DMSO-*d*_6_): *δ* 173.30, 172.41, 165.83, 156.06, 148.80, 147.52, 139.77, 139.69, 139.06, 135.99, 135.86, 131.83, 131.37, 128.27, 127.24, 127.02, 126.44, 126.35, 111.14, 52.31, 49.21, 18.41, 18.14, 17.51. ESI-HRMS calcd. For C_24_H_22_BrCl_2_N_6_O_5_: [M−H]^−^ 623.0212, found 623.0209.

##### *Methyl{2*-*[3*-*bromo*-*1*-*(3*-*chloropyridin*-*2*-*yl)*-*1H*-*pyrazole*-*5*-*carboxamido)*-*5*-*chloro*-*3*-*methylbenzoyl)*-*l*-*alanyl*-*l*-*methioninate* (4 h)

White solid in 65% yield; ^1^H NMR (600 MHz, DMSO-*d*_6_): *δ* 10.25 (s, 1H), 8.48 (dd, *J* = 4.7, 1.5 Hz, 1H), 8.40 (d, *J* = 7.0 Hz, 1H), 8.31 (d, *J* = 8.7 Hz, 1H), 8.16 (dd, *J* = 8.1, 1.5 Hz, 1H), 7.61 (dd, *J* = 8.1, 4.7 Hz, 1H), 7.54 (d, *J* = 2.5 Hz, 1H), 7.49 (d, *J* = 2.4 Hz, 1H), 7.37 (s, 1H), 4.42–4.37 (m, 1H), 4.30 (q, *J* = 7.2 Hz, 1H), 3.62 (s, 3H), 2.48–2.36 (m, 2H), 2.17 (s, 3H), 2.00 (s, 3H), 1.98–1.86 (m, 2H), 1.21 (d, *J* = 7.2 Hz, 3H). ^13^C NMR (151 MHz, DMSO-*d*_6_): *δ* 172.93, 172.58, 165.95, 156.10, 148.80, 147.53, 139.70, 139.68, 139.07, 135.95, 131.85, 131.74, 131.44, 128.30, 127.28, 127.06, 126.39, 111.09, 52.45, 51.16, 49.54, 31.59, 30.91, 18.35, 18.09, 15.04. ESI-HRMS calcd. For C_26_H_26_BrCl_2_N_6_O_5_S: [M−H]^−^ 683.0246, found 683.0237.

##### *Dimethyl{2*-*[3*-*bromo*-*1*-*(3*-*chloropyridin*-*2*-*yl)*-*1H*-*pyrazole*-*5*-*carboxamido)*-*5*-*chloro*-*3*-*methylbenzoyl)*-*l*-*alanyl*-*l*-*aspartate* (4i)

White solid in 64% yield; ^1^H NMR (600 MHz, DMSO-*d*_6_): *δ* 10.21 (s, 1H), 8.47 (d, *J* = 4.7 Hz, 1H), 8.38 (d, *J* = 7.4 Hz, 1H), 8.33 (d, *J* = 8.0 Hz, 1H), 8.15 (dd, *J* = 8.1, 1.5 Hz, 1H), 7.60 (dd, *J* = 8.1, 4.7 Hz, 1H), 7.48 (d, *J* = 2.4 Hz, 1H), 7.45 (dd, *J* = 7.3, 2.5 Hz, 1H), 7.36 (d, *J* = 3.0 Hz, 1H), 4.64 (q, *J* = 6.6 Hz, 1H), 4.31 (q, *J* = 7.3 Hz, 1H), 3.61 (s, 3H), 3.58 (d, *J* = 9.6 Hz, 3H), 2.80 (dd, *J* = 16.4, 6.2 Hz, 1H), 2.70 (dd, *J* = 16.5, 6.9 Hz, 1H), 2.16 (s, 3H), 1.17 (d, *J* = 7.4 Hz, 3H). ^13^C NMR (151 MHz, DMSO-*d*_*6*_): *δ* 172.59, 171.43, 170.92, 170.88, 165.88, 156.05, 148.83, 147.53, 139.68, 139.09, 135.95, 131.84, 131.77, 131.39, 128.32, 127.24, 127.05, 126.35, 111.10, 52.68, 52.14, 49.02, 48.98, 35.96, 18.10, 18.01. ESI-HRMS calcd. For C_26_H_24_BrCl_2_N_6_O_7_: [M−H]^−^ 681.0267, found 681.0278.

##### *Methyl{2*-*[3*-*bromo*-*1*-*(3*-*chloropyridin*-*2*-*yl)*-*1H*-*pyrazole*-*5*-*carboxamido)*-*5*-*chloro*-*3*-*methylbenzoyl)*-*l*-*alanyl*-*l*-*phenylalaninate* (4j)

White solid in 56% yield; ^1^H NMR (600 MHz, DMSO-*d*_6_): *δ* 10.22 (s, 1H), 8.48 (d, *J* = 4.6 Hz, 1H), 8.30 (t, *J* = 8.3 Hz, 2H), 8.15 (d, *J* = 7.9 Hz, 1H), 7.60 (dd, *J* = 6.7, 4.7 Hz, 1H), 7.49 (s, 1H), 7.38 (d, *J* = 4.0 Hz, 2H), 7.24 (t, *J* = 7.7 Hz, 2H), 7.20 (d, *J* = 7.6 Hz, 3H), 4.47 (q, *J* = 7.4 Hz, 1H), 4.36 (q, *J* = 7.3 Hz, 1H), 3.58 (s, 3H), 3.02 (dd, *J* = 13.9, 5.8 Hz, 1H), 2.94 (dd, *J* = 13.9, 8.6 Hz, 1H), 2.17 (s, 3H), 1.15 (dd, *J* = 7.1, 1.5 Hz, 3H). ^13^C NMR (151 MHz, DMSO-*d*_6_): *δ* 173.42, 173.30, 172.46, 172.35, 165.61, 156.31, 156.00, 148.85, 147.52, 139.66, 136.05, 131.78, 131.37, 130.56, 128.34, 127.88, 127.22, 127.03, 126.28, 115.41, 115.33, 111.09, 54.28, 54.03, 49.03, 36.63, 21.52, 18.48. ESI-HRMS calcd. For C_30_H_26_BrCl_2_N_6_O_5_: [M−H]^−^ 699.0525, found 699.0523.

##### *Methyl{2*-*[3*-*bromo*-*1*-*(3*-*chloropyridin*-*2*-*yl)*-*1H*-*pyrazole*-*5*-*carboxamido]*-*5*-*chloro*-*3*-*methylbenzoyl}*-*l*-*alanyl*-*l*-*tyrosinate* (4k)

White solid in 66% yield; ^1^H NMR (600 MHz, DMSO-*d*_6_): *δ* 10.23 (s, 1H), 9.20 (d, *J* = 5.6 Hz, 1H), 8.47 (s, 1H), 8.31 (s, 1H), 8.31–8.27 (m, 1H), 8.23 (d, *J* = 7.4 Hz, 1H), 8.15 (dd, *J* = 8.0, 1.5 Hz, 1H), 7.60 (dd, *J* = 8.1, 4.7 Hz, 1H), 7.49 (s, 1H), 7.42 (dd, *J* = 15.3, 2.5 Hz, 1H), 7.37 (d, *J* = 4.5 Hz, 1H), 6.98 (d, *J* = 8.1 Hz, 2H), 6.63 (t, *J* = 8.6 Hz, 2H), 4.45–4.28 (m, 2H), 3.58 (d, *J* = 23.1 Hz, 3H), 2.95–2.75 (m, 2H), 2.17 (d, *J* = 2.9 Hz, 3H), 1.10 (dd, *J* = 60.6, 7.1 Hz, 3H). ^13^C NMR (151 MHz, DMSO-*d*_6_): *δ* 173.42, 173.30, 172.46, 172.35, 165.60, 156.31, 156.00, 148.85, 147.52, 139.67, 139.05, 136.05, 131.78, 131.37, 130.56, 128.34, 127.22, 127.03, 126.28, 115.41, 115.33, 111.09, 54.28, 54.03, 49.03, 36.63, 21.52, 18.11. ESI-HRMS calcd for C_30_H_26_BrCl_2_N_6_O_6_: [M−H]^−^ 715.0474, found 715.0474.

#### Synthesis of compound 5a–k

Compounds **5a**–**k** were prepared with similar hydrolysis reactions as used to prepare compounds **3a** and **3b**.

##### *{2*-*[3*-*Bromo*-*1*-*(3*-*chloropyridin*-*2*-*yl)*-*1H*-*pyrazole*-*5*-*carboxamido)*-*5*-*chloro*-*3*-*methylbenzoyl}glycylglycine* (5a)

White solid in 90% yield; ^1^H NMR (600 MHz, DMSO-*d*_6_): *δ* 10.26 (d, *J* = 4.6 Hz, 1H), 8.64 (d, *J* = 5.5 Hz, 1H), 8.49 (s, 1H), 8.16 (t, *J* = 6.3 Hz, 1H), 8.11 (d, *J* = 5.6 Hz, 1H), 7.61 (dd, *J* = 8.4, 4.5 Hz, 1H), 7.53 (s, 1H), 7.50 (s, 1H), 7.33 (d, *J* = 4.6 Hz, 1H), 3.80–3.74 (m, 4H), 2.17 (d, *J* = 4.6 Hz, 3H). ^**1**3^C NMR (151 MHz, DMSO-*d*_*6*_): *δ* 171.51, 169.47, 166.66, 156.24, 148.73, 147.56, 139.90, 139.77, 139.16, 135.72, 131.96, 131.38, 128.19, 127.29, 127.05, 126.24, 111.19, 42.88, 41.05, 40.38, 18.13. ESI-HRMS calcd. For C_21_H_16_BrCl_2_N_6_O_5_: [M−H]^−^ 580.9743, found 580.9753.

##### *{2*-*[3*-*Bromo*-*1*-*(3*-*chloropyridin*-*2*-*yl)*-*1H*-*pyrazole*-*5*-*carboxamido)*-*5*-*chloro*-*3*-*methylbenzoyl)glycyl*-*l*-*alanine* (5b)

White solid in 85% yield; ^1^H NMR (600 MHz, DMSO-*d*_6_): *δ* 12.58 (s, 1H), 10.24 (s, 1H), 8.52 (t, *J* = 6.0 Hz, 1H), 8.49 (d, *J* = 4.7 Hz, 1H), 8.17 (dd, *J* = 11.7, 7.8 Hz, 2H), 7.60 (dd, *J* = 8.2, 4.8 Hz, 1H), 7.48 (d, *J* = 15.4 Hz, 2H), 7.34 (s, 1H), 4.24 (q, *J* = 7.3 Hz, 1H), 3.85–3.73 (m, 2H), 2.16 (s, 3H), 1.27 (d, *J* = 7.2 Hz, 3H). ^13^C NMR (151 MHz, DMSO-*d*_6_): *δ* 172.55, 172.48, 172.31, 165.79, 165.72, 156.03, 148.84, 147.53, 139.68, 139.16, 136.15, 131.77, 131.41, 128.33, 127.22, 127.04, 126.31, 111.14, 54.96, 49.05, 18.33, 18.11. ESI-HRMS calcd. For C_22_H_18_BrCl_2_N_6_O_5_: [M−H]^−^ 594.9899, found 594.9901.

##### *{2*-*[3*-*Bromo*-*1*-*(3*-*chloropyridin*-*2*-*yl)*-*1H*-*pyrazole*-*5*-*carboxamido)*-*5*-*chloro*-*3*-*methylbenzoyl)glycyl*-*l*-*leucine* (5c)

White solid in 91% yield; ^1^H NMR (600 MHz, DMSO-*d*_6_): *δ* 12.56 (s, 1H), 10.26 (s, 1H), 8.52 (t, *J* = 5.9 Hz, 1H), 8.49 (dd, *J* = 4.7, 1.5 Hz, 1H), 8.17–8.13 (m, 2H), 7.60 (dd, *J* = 8.1, 4.7 Hz, 1H), 7.50 (d, *J* = 2.5 Hz, 1H), 7.48 (d, *J* = 2.5 Hz, 1H), 7.36 (s, 1H), 4.30–4.22 (m, 1H), 3.83 (dd, *J* = 16.6, 6.0 Hz, 1H), 3.76 (dd, *J* = 16.6, 5.8 Hz, 1H), 2.16 (s, 3H), 1.66–1.58 (m, 1H), 1.54–1.50 (m, 2H), 0.87 (d, *J* = 6.6 Hz, 3H), 0.83 (d, *J* = 6.5 Hz, 3H). ^13^C NMR (151 MHz, DMSO-*d*_6_): *δ* 174.38, 169.00, 166.45, 156.14, 148.75, 147.53, 139.85, 139.74, 139.28, 135.75, 132.01, 131.94, 131.38, 128.20, 127.27, 127.02, 126.17, 111.18, 50.70, 42.60, 40.55, 24.75, 23.29, 21.85, 18.16. ESI-HRMS calcd. For C_25_H_24_BrCl_2_N_6_O_5_: [M−H]^−^ 637.0369, found 637.0383.

##### *{2*-*[3*-*Bromo*-*1*-*(3*-*chloropyridin*-*2*-*yl)*-*1H*-*pyrazole*-*5*-*carboxamido)*-*5*-*chloro*-*3*-*methylbenzoyl)glycyl*-*l*-*valine* (5d)

White solid in 89% yield; ^1^H NMR (600 MHz, DMSO-*d*_*6*_): *δ* 10.31 (s, 1H), 8.34 (dd, *J* = 4.8, 1.6 Hz, 1H), 7.91 (dd, *J* = 8.1, 1.6 Hz, 1H), 7.82 (s, 1H), 7.40–7.35 (m, 2H), 7.29 (d, *J* = 2.3 Hz, 1H), 7.18 (d, *J* = 9.0 Hz, 1H), 7.00 (s, 1H), 4.31 (dd, *J* = 9.0, 4.7 Hz, 1H), 4.25 (dd, *J* = 15.2, 6.7 Hz, 1H), 3.68 (dd, *J* = 15.1, 5.3 Hz, 1H), 2.18 (s, 3H), 2.15–2.07 (m, *J* = 4.9 Hz, 1H), 0.89 (d, *J* = 6.9 Hz, 3H), 0.80 (d, *J* = 6.9 Hz, 3H). ^13^C NMR (151 MHz, DMSO-*d*_6_): *δ* 174.38, 169.00, 166.45, 156.14, 148.75, 147.53, 139.85, 139.74, 139.28, 135.75, 132.01, 131.94, 131.38, 128.20, 127.27, 127.02, 126.17, 111.18, 50.70, 42.60, 24.75, 23.29, 21.85, 18.16. ESI-HRMS calcd. For C_24_H_22_BrCl_2_N_6_O_5_: [M−H]^−^ 623.0212, found 623.0212.

##### *{2*-*[3*-*Bromo*-*1*-*(3*-*chloropyridin*-*2*-*yl)*-*1H*-*pyrazole*-*5*-*carboxamido)*-*5*-*chloro*-*3*-*methylbenzoyl)glycyl*-*l*-*serine* (5e)

White solid in 87% yield; ^**1**^**H NMR** (600 MHz, DMSO-*d*_6_) *δ* 10.24 (s, 1H), 8.49 (d, *J* = 4.1 Hz, 1H), 8.16 (d, *J* = 8.1 Hz, 1H), 8.10 (d, *J* = 7.9 Hz, 1H), 7.60 (dd, *J* = 8.2, 4.7 Hz, 1H), 7.50 (s, 1H), 7.45 (s, 1H), 7.36 (s, 1H), 4.31 (dt, *J* = 8.4, 4.7 Hz, 1H), 3.85 (qd, *J* = 16.7, 6.0 Hz, 2H), 3.71 (dd, *J* = 11.0, 5.0 Hz, 1H), 3.62 (dd, *J* = 11.0, 4.2 Hz, 1H), 2.16 (s, 3H). ^13^C NMR (151 MHz, DMSO-*d*_6_): *δ* 171.51, 169.47, 166.66, 156.24, 148.73, 147.56, 139.91, 139.77, 139.16, 135.72, 131.96, 131.38, 128.19, 127.29, 127.05, 126.24, 111.19, 42.88, 41.05, 40.38, 18.13. ESI-HRMS calcd. For C_22_H_18_BrCl_2_N_6_O_6_: [M−H]^−^ 610.9848, found 610.9845.

##### *{2*-*[3*-*Bromo*-*1*-*(3*-*chloropyridin*-*2*-*yl)*-*1H*-*pyrazole*-*5*-*carboxamido)*-*5*-*chloro*-*3*-*methylbenzoyl}*-*l*-*alanylglycine* (5f)

White solid in 93% yield; ^1^H NMR (600 MHz, DMSO-*d*_6_): *δ* 10.24 (s, 1H), 8.48 (dd, *J* = 4.8, 1.5 Hz, 1H), 8.46 (d, *J* = 7.3 Hz, 1H), 8.16 (dd, *J* = 8.1, 1.5 Hz, 1H), 8.10 (t, *J* = 5.9 Hz, 1H), 7.60 (dd, *J* = 8.1, 4.7 Hz, 1H), 7.51 (dd, *J* = 19.1, 2.4 Hz, 2H), 7.35 (s, 1H), 4.31 (q, *J* = 7.2 Hz, 1H), 3.73 (d, *J* = 5.9 Hz, 2H), 2.18 (s, 3H), 1.20 (d, *J* = 7.2 Hz, 3H). ^13^C NMR (151 MHz, DMSO-*d*_6_): *δ* 172.55, 172.48, 172.31, 165.79, 165.72, 156.03, 148.84, 147.53, 139.68, 139.16, 136.15, 131.77, 131.41, 128.33, 127.22, 127.04, 126.31, 111.14, 54.96, 49.05, 18.33, 18.11. ESI-HRMS calcd. For C_22_H_18_BrCl_2_N_6_O_5_: [M−H]^−^ 594.9899, found 594.9896.

##### *{2*-*[3*-*Bromo*-*1*-*(3*-*chloropyridin*-*2*-*yl)*-*1H*-*pyrazole*-*5*-*carboxamido]*-*5*-*chloro*-*3*-*methylbenzoyl}*-*l*-*alanyl*-*l*-*alanine* (5g)

White solid in 91% yield; ^1^H NMR (600 MHz, DMSO-*d*_*6*_): *δ* 10.25 (s, 1H), 8.48 (dd, *J* = 4.7, 1.5 Hz, 1H), 8.36 (d, *J* = 7.4 Hz, 1H), 8.16 (dd, *J* = 8.1, 1.6 Hz, 1H), 8.13 (d, *J* = 7.5 Hz, 1H), 7.60 (dd, *J *= 8.1, 4.7 Hz, 1H), 7.49 (s, 2H), 7.36 (s, 1H), 4.34 (q, *J* = 7.2 Hz, 1H), 4.21 (q, *J* = 7.3 Hz, 1H), 2.17 (s, 3H), 1.24 (d, *J* = 7.3 Hz, 3H), 1.19 (d, *J* = 7.2 Hz, 3H). ^13^C NMR (151 MHz, DMSO-*d*_6_): *δ* 174.39, 172.20, 165.79, 156.03, 148.80, 147.53, 139.74, 139.69, 139.06, 135.95, 131.83, 131.81, 131.37, 128.29, 127.23, 127.04, 126.39, 111.10, 49.19, 47.92, 18.44, 18.11, 17.77. ESI-HRMS calcd. For C_23_H_20_BrCl_2_N_6_O_5_: [M−H]^−^ 609.0056, found 609.0041.

##### *{2*-*[3*-*Bromo*-*1*-*(3*-*chloropyridin*-*2*-*yl)*-*1H*-*pyrazole*-*5*-*carboxamido)*-*5*-*chloro*-*3*-*methylbenzoyl)*-*l*-*alanyl*-*l*-*methionine* (5h)

White solid in 86% yield; ^1^H NMR (600 MHz, DMSO-*d*_6_): *δ* 12.58 (s, 1H), 10.22 (s, 1H), 8.48 (d, *J* = 4.8 Hz, 1H), 8.37 (d, *J* = 7.4 Hz, 1H), 8.15 (t, *J* = 8.1 Hz, 2H), 7.60 (dd, *J* = 7.9, 5.0 Hz, 1H), 7.48 (s, 1H), 7.44 (s, 1H), 7.37 (s, 1H), 4.38–4.28 (m, 2H), 2.49–2.39 (m, 2H), 2.16 (s, 3H), 2.02 (s, 3H), 2.00–1.94 (m, 1H), 1.90–1.82 (m, 1H), 1.20 (d, *J* = 7.1 Hz, 3H). ^13^C NMR (151 MHz, DMSO-*d*_6_): *δ* 173.65, 172.66, 165.87, 156.01, 148.86, 147.53, 139.68, 139.66, 139.01, 136.08, 131.77, 131.39, 130.10, 128.34, 127.24, 127.04, 126.42, 111.06, 51.13, 49.50, 31.20, 30.08, 18.45, 18.10, 15.06. ESI-HRMS calcd. For C_25_H_24_BrCl_2_N_6_O_5_S: [M−H]^−^ 669.0089, found 669.0085.

##### *{2*-*[3*-*Bromo*-*1*-*(3*-*chloropyridin*-*2*-*yl)*-*1H*-*pyrazole*-*5*-*carboxamido)*-*5*-*chloro*-*3*-*methylbenzoyl)*-*l*-*alanyl*-*l*-*aspartic acid* (5i)

White solid in 85% yield; ^1^H NMR (600 MHz, DMSO-*d*_6_): *δ* 12.54 (s, 2H), 10.21 (s, 1H), 8.48 (d, *J* = 4.8 Hz, 1H), 8.35 (t, *J* = 6.5 Hz, 1H), 8.20 (t, *J* = 6.6 Hz, 1H), 8.16 (d, *J* = 8.0 Hz, 1H), 7.60 (dd, *J* = 7.9, 4.9 Hz, 1H), 7.49 (s, 1H), 7.45 (s, 1H), 7.38 (s, 1H), 4.54 (q, *J* = 6.8 Hz, 1H), 4.35 (q, *J* = 7.1 Hz, 1H), 2.74–2.65 (m, 1H), 2.62–2.53 (m, 1H), 2.17 (s, 3H), 1.17 (t, *J* = 8.8 Hz, 3H). ^13^C NMR (151 MHz, DMSO-*d*_*6*_): *δ* 172.59, 171.43, 170.92, 170.88, 165.88, 156.05, 148.83, 147.53, 139.68, 139.09, 135.95, 131.84, 131.77, 131.39, 128.32, 127.24, 127.05, 126.35, 111.10, 52.68, 52.14, 49.02, 18.10, 18.01. ESI-HRMS calcd. For C_24_H_20_BrCl_2_N_6_O_7_: [M−H]^−^ 652.9954, found 652.9950.

##### *{2*-*[3*-*Bromo*-*1*-*(3*-*chloropyridin*-*2*-*yl)*-*1H*-*pyrazole*-*5*-*carboxamido)*-*5*-*chloro*-*3*-*methylbenzoyl)*-*l*-*alanyl*-*l*-*phenylalanine* (5j)

White solid in 79% yield; ^1^H NMR (600 MHz, DMSO-*d*_6_): *δ* 12.79 (s, 1H), 10.22 (s, 1H), 8.47 (d, *J* = 4.8 Hz, 1H), 8.22 (d, *J* = 7.5 Hz, 1H), 8.18 (d, *J* = 8.3 Hz, 1H), 8.15 (d, *J* = 8.1 Hz, 1H), 7.60 (dd, *J* = 8.1, 4.9 Hz, 1H), 7.48 (s, 1H), 7.40 (d, *J* = 2.6 Hz, 1H), 7.37 (s, 1H), 7.24–7.16 (m, 5H), 4.46 (td, *J* = 9.0, 4.5 Hz, 1H), 4.32 (q, *J* = 7.2 Hz, 1H), 3.08 (dd, *J* = 13.8, 4.7 Hz, 1H), 2.87 (dd, *J* = 13.7, 9.6 Hz, 1H), 2.16 (s, 3H), 1.01 (d, *J* = 7.1 Hz, 3H). ^13^C NMR (151 MHz, DMSO-*d*_6_): *δ* 173.30, 172.38, 165.59, 156.00, 148.85, 147.52, 139.67, 139.05, 137.89, 136.01, 131.79, 131.35, 129.66, 129.63, 128.58, 128.52, 128.34, 127.22, 127.04, 126.84, 126.31, 111.09, 53.67, 49.07, 37.34, 18.41, 18.11. ESI-HRMS calcd. For C_29_H_24_BrCl_2_N_6_O_5_: [M−H]^−^ 685.0369, found 685.0366.

##### *{2*-*[3*-*Bromo*-*1*-*(3*-*chloropyridin*-*2*-*yl)*-*1H*-*pyrazole*-*5*-*carboxamido]*-*5*-*chloro*-*3*-*methylbenzoyl}*-*l*-*alanyl*-*l*-*tyrosine* (5k)

White solid in 88% yield; ^1^H NMR (600 MHz, DMSO-*d*_*6*_): *δ* 12.68 (s, 1H), 10.24 (s, 1H), 9.18 (s, 1H), 8.48 (dd, *J* = 4.6, 1.5 Hz, 1H), 8.31 (s, 1H), 8.15 (d, *J* = 8.1 Hz, 1H), 8.07 (dd, *J* = 63.5, 8.0 Hz, 1H), 7.60 (dd, *J* = 8.1, 4.7 Hz, 1H), 7.49 (s, 1H), 7.41 (dd, *J* = 11.6, 2.5 Hz, 1H), 7.38 (d, *J* = 5.1 Hz, 1H), 6.99 (dd, *J* = 8.3, 4.9 Hz, 2H), 6.62 (t, *J* = 8.3 Hz, 2H), 4.39–4.30 (m, 2H), 2.91 (dd, *J* = 13.9, 5.3 Hz, 1H), 2.78 (ddd, *J* = 36.8, 13.9, 8.8 Hz, 1H), 2.16 (s, 3H), 1.09 (dd, *J* = 65.8, 7.1 Hz, 3H). ^13^C NMR (151 MHz, DMSO-*d*_6_): *δ* 173.27, 172.45, 165.78, 156.31, 156.08, 148.81, 147.52, 139.67, 139.13, 136.08, 131.81, 131.67, 130.54, 128.31, 127.81, 127.26, 127.05, 126.24, 115.44, 115.36, 111.12, 79.55, 54.28, 54.04, 49.07, 40.39, 18.15, 18.09. ESI-HRMS calcd. For C_29_H_24_BrCl_2_N_6_O_6_: [M−H]^−^ 701.0318, found 701.0311.

### Determination of phloem mobility

#### Plant materials

Castor bean seeds (*Ricinus communis* L.) No. 9 were purchased from the Agricultural Science Academy (Zibo, China) and nurtured in wet absorbent cotton for 24 h at 27 ± 1 °C prior to sowing in vermiculite watered with tap water. Seedlings were grown at 27 ± 1 °C and 80% ± 5% RH during the photoperiod (14 h) for 6 days.

#### Screening of phloem mobility

The already described procedure [30] was utilized to the phloem sap collection. After endosperms being carefully stripped off, cotyledons were incubated in buffered solution containing 20 mM 4-morpholineethanesulfonic acid (MES) (pH = 5.0), 0.25 mM MgCl_2_, and 0.5 mM CaCl_2_ for 0.5 h, then immediately transferred to a new MES culture fluid containing 100 μM of the test compounds. After the cotyledons were incubated for 2 h, the hypocotyls were severed in the hook region for phloem exudation, and the phloem sap was continually collected for 2 h.

#### Analytical methods

After dilution with methanol (phloem sap/methanol = 1:4, v/v), the collected phloem sap was analyzed on high-performance liquid chromatograph (HPLC, Agilent 1290 series) equipped with a UV detector at 210 nm. Separations were done on a SB-C_18_ reversed-phase column (5 μm, 250 × 4.6 mm i.d.; Agilent) with a flow rate of 1.0 mL/min at 30 °C, and the eluent was made of acetonitrile and water containing 0.1% trifluoroethanoic acid (TFA). The calibration curves of the test compounds (ranging from 0 to 100 μM) were linear.

### Calculation of physicochemical properties

The physicochemical properties of all conjugates and chlorantraniliprole, including ionization constant in aqueous solution (p*K*_a_) and octanol/water partitioning coefficient (log *K*_*o/w*_), were predicted by ACD LogD software suite version 14.0 [31].

### Biological assay in vitro

The insecticidal activities of compounds **4g** and **5g** against *Plutella xylostella* L. and *Spodoptera exigua* were tested according to literature [6], and the insecticidal activity of chlorantraniliprole was also tested as positive control. Leaf-disc dipping assays were employed with petri dishes kept in incubator at 26 °C and 85% relative humidity under a photoperiod of 16 h:8 h (light/dark). Mortalities were checked after treated for 48 h.

## Results and discussion

### Phloem mobility in *R. communis*

Phloem mobilities of all dipeptide conjugates were studied in *R. communis*, which is an ideal model to investigate the phloem mobility of xenobiotics [10, 12, 32]. An interesting observation was that the methyl ester structure on compounds **4a**–**k** were mostly hydrolyzed to their corresponding acidic conjugates during the uptake and transport process in planta. When *R. communis* cotyledons were treated with dipeptide methyl ester conjugates **4a**–**k**, the concentrations of tested compound in phloem sap were close to the limit of detection, and only corresponding dipeptide conjugates **5a**–**k** were detected by HPLC. Therefore, the phloem mobility of the dipeptide methyl ester conjugates **4a**–**k** was represented by the amount of corresponding dipeptide conjugates being detected.

**Fig. 1:**
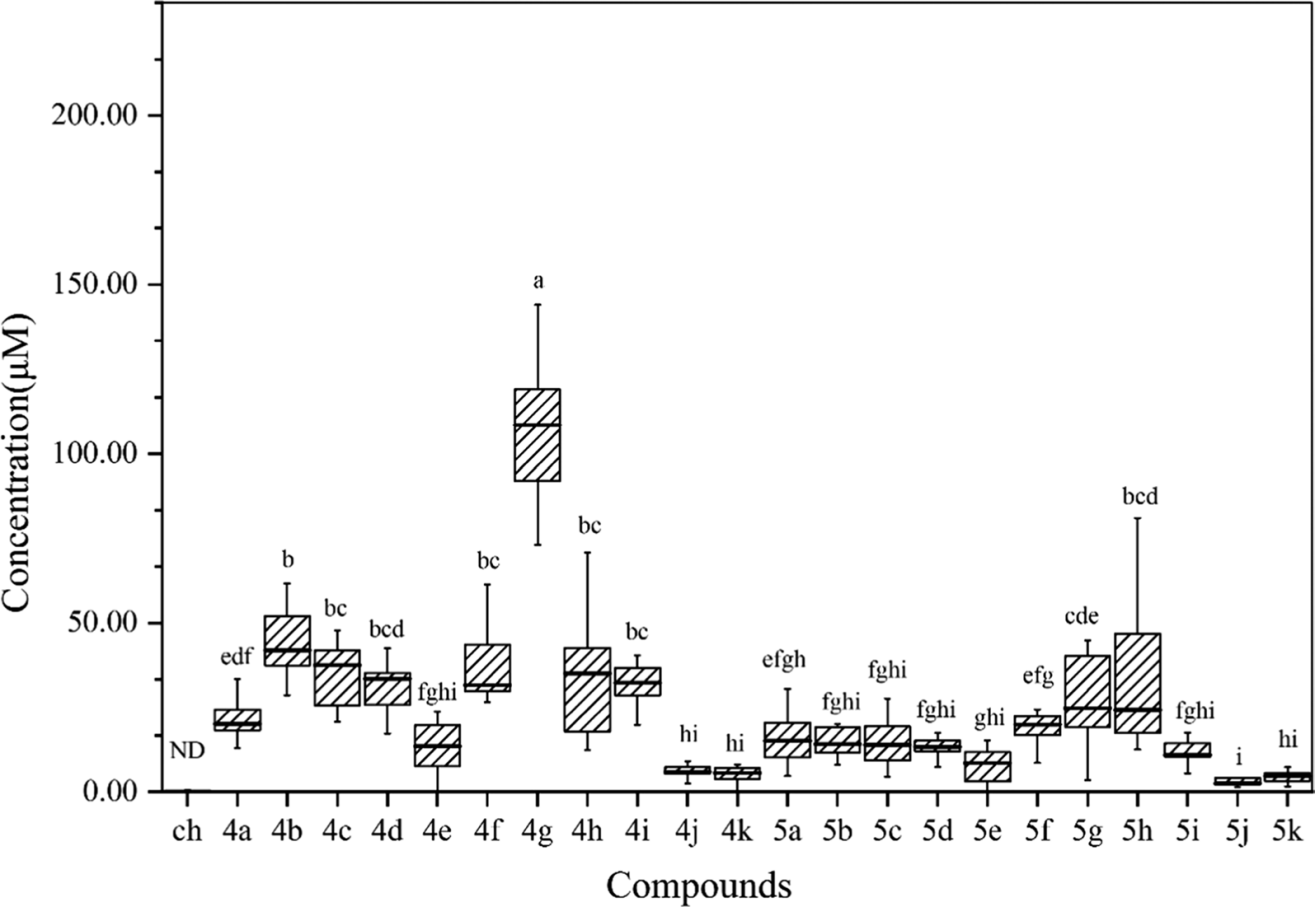
Concentrations of different dipeptide conjugates in phloem sap of *Ricinus communis*. Cotyledons were incubated in incubation solution contain 100 μM of tested compound. The phloem sap collection started 30 min after pre-incubation and lasted for 2 h. Duncan’s multiple-range tests at a 5% probability level were used to determine statistical differences among treatments (*P* < 0.05). *Ch* chlorantraniliprole, *ND* not detected. The data (mean ± SE, n = 10) with a column topped by different letters are significantly different from each other

Results from systemicity tests demonstrated that the non-phloem-mobile chlorantraniliprole could acquire phloem mobility by conjugating with dipeptide or dipeptide methyl ester structures. As shown in Fig. 1, both the dipeptide methyl ester conjugates **4a**–**k** and corresponding dipeptide conjugates **5a**–**k** were able to be absorbed by cotyledons of *R. communis*, while the parent insecticide chlorantraniliprole could not be detected in the phloem sap. Especially, the concentrations of compounds **4a**–**k** in phloem sap (in forms of hydrolysis products) were generally higher than that of corresponding dipeptide conjugates **5a**–**k**. In particular, compound **4g** represented the highest concentration (114.49 ± 11.10 μM) under the existing experimental conditions in the form of its hydrolysis product **5g**, which has exceeded its concentration in incubation solution (100 μM). There was no significant difference between the detected concentrations of **4b**, **4c**, **4d**, **4f**, **4h**, **4i**, all of which were significantly lower than that of conjugate **4g**.

In addition to the difference between acids and esters, the side chain groups on dipeptide residues also showed effects on phloem mobility of dipeptide-chlorantraniliprole conjugates. By comparing the difference between the uptake amounts of the conjugates, it was found that the substituent R at the α position in the amino acid structure was related to the transport of the conjugate in plants. When R = methyl, the conjugate showed the best phloem mobility. When the size of substituent group gradually increase, the phloem mobility of the conjugate would decrease. The conjugates carrying aromatic rings on the side chain, such as **4j** and **4k**, exhibited the lowest concentration in the phloem sap. It demonstrated that the dipeptides with aromatic ring on the side chain was less effective on improving the phloem uptake.

### Predicted phloem mobility

Our previous research indicated that the phloem mobility of pesticide-nutrient conjugates could be influenced by their physicochemical properties and affinities with active carrier systems, which can affect the efficiency of passive diffusion and active transportation respectively [6, 8, 11]. In order to further interpret the structure-phloem mobility relationship of dipeptide-chlorantraniliprole conjugates, their physicochemical properties were first calculated and analyzed. For each conjugate, ionization constant in aqueous solution (p*K*_a_) and octanol/water partitioning coefficient (log *K*_o/w_) were predicted (Table 1).

**Table 1:** Molecular weights, log *K*_*o/w*_, and p*K*_a_ of compounds **4a**–**5k**

Compounds	Molecular weight (g/mol)	Log *K*_*o/w*_	p*K*_a_
4a	598.23	3.48	10.33 ± 0.7
4b	612.26	3.72	10.33 + 0.7
4c	654.34	4.5	10.33 + 0.7
4d	640.31	4.28	10.33 + 0.7
4e	628.26	3.04	10.33 ± 0.7
4f	612.26	3.72	10.33 ± 0.7
4g	626.29	4.06	10.33 ± 0.7
4h	686.4	4.6	10.33 ± 0.7
4i	684.32	4.47	10.33 ± 0.7
4j	702.38	5.94	10.33 ± 0.7
4k	718.38	5.21	9.75 ± 0.15
5a	584.21	1.04	3.29 ± 0.1
5b	598.23	1.37	3.38 ± 0.1
5c	640.31	2.42	3.36 ± 0.1
5d	626.29	1.88	3.32 ± 0.1
5e	614.23	0.3	2.97 ± 0.1
5f	598.23	1.29	3.3 ± 0.1
5g	612.26	1.71	3.39 ± 0.1
5h	672.38	2.26	3.21 ± 0.1
5i	656.27	0.02	2.85 ± 0.1
5j	688.36	3.06	3.49 ± 0.1
5k	704.36	2.29	3.07 ± 0.1

**Fig. 2:**
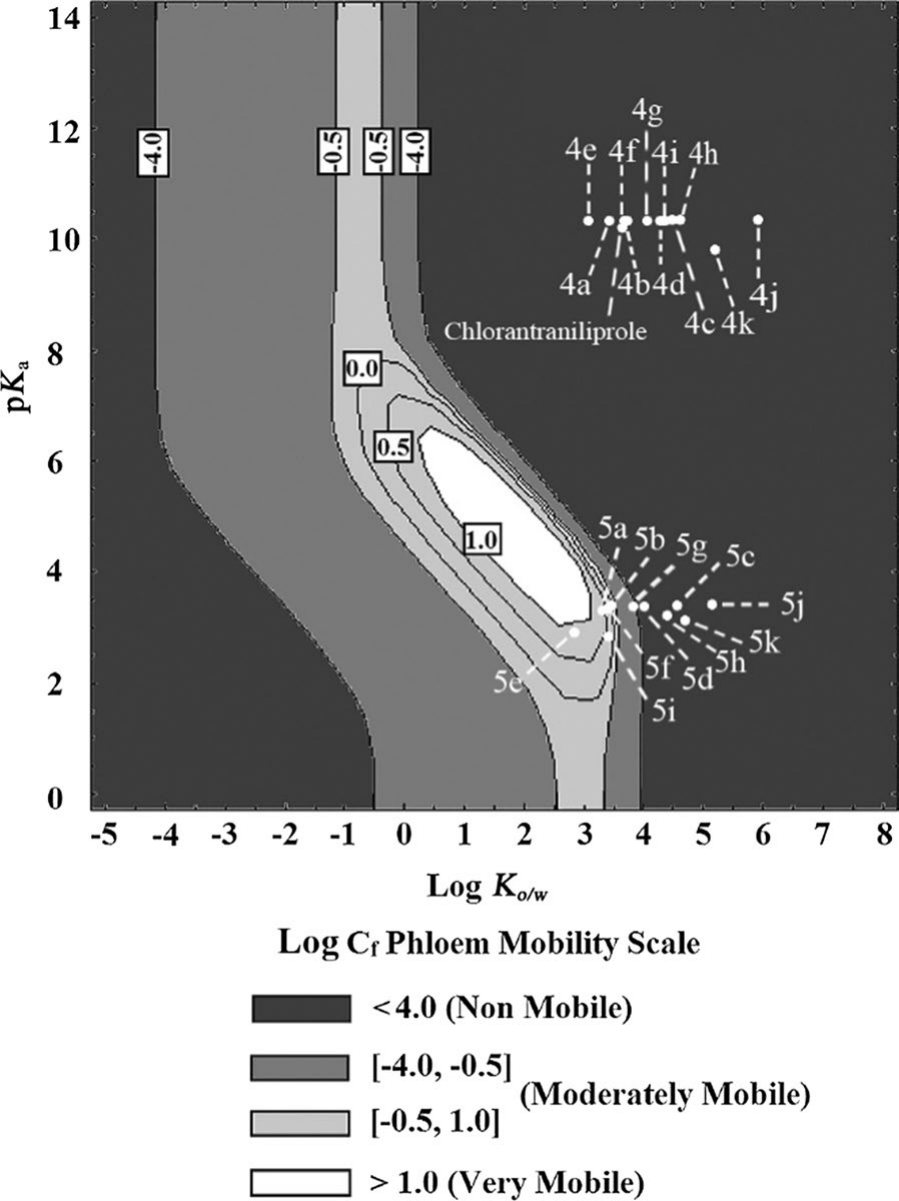
Predication of phloem mobility of dipeptide conjugates via the Kleier map (log *C*_f_ as a function of log *K*_*o/w*_ and p*K*_a_). Log *K*_*o/w*_ and p*K*_a_ were calculated by ACD LogD suite v.14.0 software

Results showed no obvious correlation between phloem systemicity and log *K*_o/w_ values. Compared to the significant differences among phloem mobilities of conjugates, the range fluctuation of log *K*_o/w_ values was small. The p*K*_a_ and log *K*_o/w_ values were then fitted in Kleier model, which was widely applied to predict the phloem mobility of xenobiotics [33, 34]. As shown in Fig. 2, the data points for all dipeptide ester conjugates **4a**–**k** were located in non-phloem-mobile area, which was not consistent with the experimental data. It was speculated that dipeptide derivatives were loaded into the phloem of plants via pathways other than passive diffusion, possibly with the participation of dipeptide transporters [35]. Although some of the dipeptide acid conjugates (**5a**, **5b**, **5e**, **5g**, **5f**, and **5i**) showed data points located in phloem-mobile area, their phloem uptake was not significantly improved compared with the ones located in non-phloem-mobile area. This indicated that passive diffusion could help the conjugates to penetrate into phloem tissue [11], but was not the major route for the uptake process. In summary, active transport may play a dominant role in the uptake and transport of dipeptide conjugates. It is possible that by linking dipeptide fragments with pesticide structure, the conjugates will be able to bind with dipeptide transporters, and thus process phloem mobility.

### Insecticidal activity

Insecticidal activities of compound **4g**, which represented the highest phloem mobility in *R. communis*, and its hydrolysis product **5g** were tested against *Plutella xylostella* L. and *Spodoptera exigua* according to methods reported in literature [36]. The insecticidal activity of chlorantraniliprole was also tested as positive control. As shown in Table 2, both compounds **4g** and **5g** maintained good biological activity against *Plutella xylostella* L. and *Spodoptera exigua* comparable to chlorantraniliprole. Compared to compound **4g**, compound **5g** showed significantly better control effect against *Plutella xylostella* L. and similar insecticidal activity against *Spodoptera exigua*. Considering the compound **4g** could be hydrolyzed into the compound **5g** and accumulate in phloem sap during the transportation process in planta, application of the ester conjugate **4g** could potentially decrease the usage of pesticides while showing similar control effect on sucking pests compared to commercial chlorantraniliprole.

**Table 2:** Insecticidal activities of compounds **4g**, **5g** and chlorantraniliprole (50 mg/L) against *Plutella xylostella* L. and *Spodoptera exigua* in 48 h

Compounds	Mortality (%)	
	*Plutella xylostella* L.	*Spodoptera exigua*
**4g**	54.58 ± 3.03^a^	76.21 ± 7.63^a^
**5g**	87.58 ± 2.89^b^	83.57 ± 4.46^ab^
Chlorantraniliprole	91.67 ± 4.81^b^	97.22 ± 2.78^b^

Test concentration was 50 mg/L. Duncan tests at a 5% probability level were used to determine statistical differences among treatments (*P *< 0.05)

## Conclusion

In summary, 22 new dipeptide-chlorantraniliprole conjugates were synthesized via simple synthetic route. All derivatives have acquired phloem mobility in *R. communis* compared to non-phloem-mobile chlorantraniliprole, which proved that dipeptides could be another option for promoiety when designing new phloem-mobile pesticides. The uptaken amounts of most dipeptide ester conjugates in phloem were better than that of corresponding dipeptide conjugates, while the dipeptide esters would be hydrolyzed to corresponding dipeptides during the uptake or transportation process. In particular, compound **4g** that conjugated with alanyl-alanine dipeptide fragment showed the highest phloem mobility among all conjugates, and could lead to high concentration of **5g** in phloem sap which exceeded its concentration in incubation solution. Bioassay results showed that the control effects of **4g** and **5g** against *Plutella xylostella* L. and *Spodoptera exigua* were comparable to that of chlorantraniliprole. Thus, application of compound **4g** could potentially lead to better in vitro control effect compared to chlorantraniliprole. Further research on the uptake mechanism of dipeptide-chlorantraniliprole conjugates and its affinity with dipeptide transporters is still in progress.

## Data Availability

The datasets used and/or analyzed during the current study are available from the corresponding author on reasonable request.

## Abbreviations

CH_2_Cl_2_: Dichloromethane
DMAP: *N*, *N*-dimethyl-4-aminopyridine
EDCI: 1-(3-Dimethylaminopropyl)-3-ethylcarbodiimide hydrochloride
HCl: Hydrochloric acid
HPLC: High-performance liquid chromatography
LiOH: Lithium hydroxide
log *K*_o/w_: Octanol/water partitioning coefficient
MES: 4-Morpholineethanesulfonic acid
NMM: *N*-methyl morpholine
p*K*_a_: Ionization constant in aqueous solution
TLC: Thin-layer chromatography
TFA: Trifluoroacetic acid
THF: Tetrahydrofuran
UV: Ultraviolet
